# Quartz Crystal Microbalance Analysis of Antimicrobial Protein Adsorption onto Zirconia

**DOI:** 10.3390/ma18163856

**Published:** 2025-08-18

**Authors:** Masatsugu Hirota, Takatsugu Yamamoto

**Affiliations:** 1 Department of Education for Dental Medicine, Tsurumi University School of Dental Medicine, 2-1-3, Tsurumi, Tsurumi-ku, Yokohama 230-8501, Japan; yamamoto-tk@tsurumi-u.ac.jp; 2 Department of Operative Dentistry, Tsurumi University School of Dental Medicine, 2-1-3, Tsurumi, Tsurumi-ku, Yokohama 230-8501, Japan

**Keywords:** QCM, protein adsorption, salivary antimicrobial protein, dental plaque, zirconia

## Abstract

Protein adsorption on dental zirconia (ZrO_2_) surfaces plays a crucial role in plaque formation, tissue healing, and bone osseointegration. This study investigated and compared the adsorption behavior of three salivary antimicrobial proteins—peroxidase, lactoferrin, and lysozyme—on a ZrO_2_ sensor and an Au sensor using a quartz crystal microbalance (QCM) operating at 27 MHz. Protein adsorption was determined from frequency decreases, and the apparent reaction rate constant (*k*_obs_) was calculated by fitting frequency–time curves to a kinetic model. The amount of lactoferrin adsorbed on the ZrO_2_ sensor was significantly higher than that of peroxidase and lysozyme (*p* < 0.05). Significantly smaller amounts of peroxidase and lysozyme were adsorbed onto the ZrO_2_ sensor than the Au sensor (*p* < 0.05). The *k*_obs_ for lysozyme on the Au sensor was significantly higher than those for lactoferrin on sensors and for peroxidase on the Au sensor (*p* < 0.05). Differences in salivary antimicrobial protein adsorption between Au and ZrO_2_ surfaces were influenced, in part, by electrostatic interactions between the proteins and the material surface.

## 1. Introduction

Over the past two decades, the application of digital technology has expanded to include partially stabilized zirconia (ZrO_2_), such as dental zirconia, in restorative and prosthetic dentistry. The CAD/CAM systems used in dentistry have enabled the clinical application of ZrO_2_ and facilitated the advancement of metal-free treatments as alternatives to metals [1,2]. By adjusting stabilizer composition and enhancing transparency, aesthetic restorations using ZrO_2_ have also become feasible [3]. In particular, ZrO_2_ is employed in dental implant components, including superstructures, abutments, and implant bodies [4]. Among white ceramics used in dental materials, ZrO_2_ offers high strength, toughness, and resistance to wear and corrosion, and it does not cause allergic reactions. ZrO_2_ is also known for its excellent biocompatibility as a biomaterial [5,6,7].

Various oral bacteria form biofilms that contribute to dental caries and periodontal diseases. Therefore, understanding plaque formation and adhesion on prosthetic devices is crucial during their development [8]. Brakel et al. [9] and Re et al. [10] investigated the in vitro growth and adhesion of oral bacteria on Ti and ZrO_2_, suggesting that plaque formation on ZrO_2_ surfaces may be lower than on Ti surfaces. Rimondini et al. [11] and Scarano et al. [12] also reported significantly lower bacterial adhesion on ZrO_2_ than on Ti in an in vivo study. This property is important in controlling inflammation in periodontal tissues and influences the long-term prognosis of dental treatments [13]. Additionally, ZrO_2_ promotes a higher proliferation rate of fibroblasts in periodontal tissues in vitro compared to Ti, and laminin-5(332) is expressed around ZrO_2_ implants in vivo [14].

Tokunaga et al. [15] and Hirota et al. [16] created a ZrO_2_ thin film on roughened Ti implant surfaces using a molecular precursor method without altering the surface topography. In rat models, ZrO_2_-coated Ti implants supported early-stage bone formation at levels equivalent to or exceeding those of non-coated Ti implants. The chemical composition of the ZrO_2_ coating was suggested to influence the orientation of collagen fiber bundles in the gingiva. Based on these findings, the mechanisms and significance of plaque formation, tissue healing, and bone integration when using ZrO_2_—compared to metallic dental materials such as Au, Ag, Co-Cr alloys, and Ti —should be further clarified.

When a material is implanted into the body, proteins are the first to adsorb onto its surface. Kasemo et al. [17] noted that these adsorbed proteins influence cell adhesion. Additionally, Trindade et al. [18] reported that osseointegration is a multi-step process that begins with protein adsorption onto dental implant surfaces. Biofilm formation in the oral cavity also proceeds in a stepwise manner, with salivary protein adsorption marking the initial phase. These adsorbed proteins on biomaterial surfaces play a key role in bacterial adhesion [19]. Therefore, investigating salivary protein adsorption on dental materials is essential for understanding the mechanisms underlying plaque formation.

The quartz crystal microbalance (QCM) technique provides a straightforward method for detecting protein adsorption onto material surfaces by measuring shifts in the oscillation frequency of a quartz sensor [20]. Yoshida et al. [21] previously used the QCM method to examine salivary protein adsorption onto various materials (Au, silica, and Ti) and identified differences in the adsorption behaviors on each surface. They further reported that Ti and stainless steel adsorbed greater amounts of lactoferrin compared to ZrO_2_ and polymethyl methacrylate [22].

Few studies have investigated the adsorption of salivary antimicrobial proteins onto ZrO_2_. Among the various antimicrobial proteins present in saliva, this study focused on three: lactoferrin, peroxidase, and lysozyme [23]. Hydrogen peroxide (H_2_O_2_) is produced by bacteria that settle on mucous membranes. Peroxidase is a key component of salivary defense, detoxifying H_2_O_2_ in the presence of thiocyanate by converting it into hypothiocyanite, dioxygen, and water [24]. Salivary peroxidase exhibits broad-spectrum antimicrobial activity against oral and non-oral bacteria, including *Streptococcus mutans*, *Fusobacterium nucleatum*, *Escherichia coli*, *Staphylococcus aureus*, and *Pseudomonas aeruginosa* [25]. Lactoferrin is an iron-binding glycoprotein that exhibits antimicrobial activity by depriving bacteria of the iron required for growth. It also shows broad antimicrobial effects against oral pathogens such as *Streptococcus mutans* and *Porphyromonas gingivalis*, as well as opportunistic pathogens like *Acinetobacter baumannii* and *Salmonella* species [26]. Lysozyme in saliva primarily targets Gram-positive bacteria such as *Staphylococcus aureus* and *Streptococcus mutans* by breaking down their cell walls [27]. However, the direct or indirect relationship between ZrO_2_ and these proteins, as well as their influence on biofilm formation and persistence, remains unclear.

In this study, we investigated the adsorption behavior of three salivary antimicrobial proteins—lactoferrin, peroxidase, and lysozyme—onto ZrO_2_ surfaces in relation to biofilm formation, using QCM analysis.

## 2. Materials and Methods

### 2.1. QCM Device and Sensors

**Figure 1:**
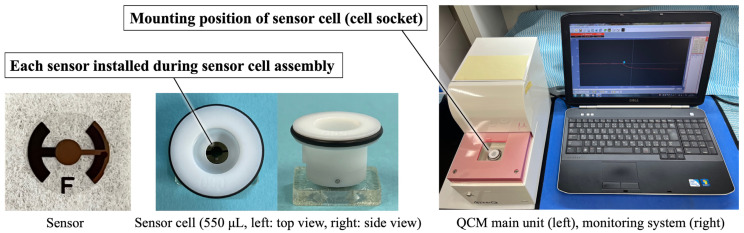
Appearance of the QCM apparatus, sensor, and sensor cell.

Figure 1 shows the QCM apparatus used in this study (27 MHz, AFFINIX QNμ, Piezo Parts Co., Ltd., Tokyo, Japan). A commercially available Au sensor (Piezo Parts Co., Ltd., Tokyo, Japan) was used as the control. The AT-cut quartz crystal, which is a quartz crystal cut at an angle of 35°15′ from the Z-axis of artificial quartz, was mounted between Au electrodes. The ZrO_2_ sensor was prepared by sputter-coating the Au electrode using sputtering deposition equipment (CS200, ULVAC, Inc., Kanagawa, Japan). A zirconium target was sputtered at 0.5 Pa in an oxygen atmosphere for 30 min. The film thickness of the sputtered ZrO_2_ was previously determined to be 115 nm using a profilometer [22,28,29,30].

The Au and ZrO_2_ sensors were assembled into sensor cells. Before QCM measurements, all sensors were exposed to ultraviolet (UV) radiation (BioForce Nanosciences Holdings Inc., Virginia Beach, VA, USA) for 20 min to remove surface contamination [31]. UV irradiation (λ = 254 nm, 15 mW/cm^2^) was applied vertically from 20 mm above. The ZrO_2_ sensor was mounted on the cell socket of the main QCM unit following UV irradiation.

### 2.2. Morphologies and Surface Roughness Values of Au and ZrO_2_ Sensors

To evaluate the three-dimensional (3D) surface topography of the Au and ZrO_2_ sensors before protein adsorption, atomic force microscopy (AFM; Nanosurf Easyscan 2, Nanosurf AG, Liestal, Switzerland) was used in air. Tapping-mode measurements were performed using a silicon probe (Budget Sensors Tap190Al-G, force constant 48 N/m, resonance frequency ~190 kHz; Innovative Solutions Bulgaria Ltd., Sofia, Bulgaria). The scanned area for AFM imaging was 5 × 5 μm^2^. The average diameter of crystalline particles on each sensor surface was determined using an image analysis system (WinROOF, Visual System Division, Mitani Corp., Tokyo, Japan). The 3D arithmetic height (Sa), representing surface roughness, was calculated over the same areas.

Five measurements were performed for each of the Au and ZrO_2_ sensors.

### 2.3. Surface Wettability of Au and ZrO_2_ Sensors

Prior to protein adsorption, the wettability of UV-cleaned Au and ZrO_2_ sensors was evaluated by measuring their contact angles using double-distilled water. A contact angle meter (DMe-201, Kyowa Interface Science Co., Ltd., Tokyo, Japan) was used. The contact angle was determined 3 s after dispensing a 0.5 µL droplet of water onto each sensor surface.

Ten measurements were performed for each of the Au and ZrO_2_ sensors at room temperature and humidity.

### 2.4. QCM Measurement and Procedure

Lactoferrin (from bovine milk), peroxidase (from horseradish), and lysozyme (from egg white) (FUJIFILM Wako Pure Chemical Corp., Osaka, Japan) were used as salivary antimicrobial proteins in this study. Each protein was dissolved in phosphate-buffered saline (PBS, pH 7.4) at a final concentration of 0.5 mg/mL.

**Figure 2:**
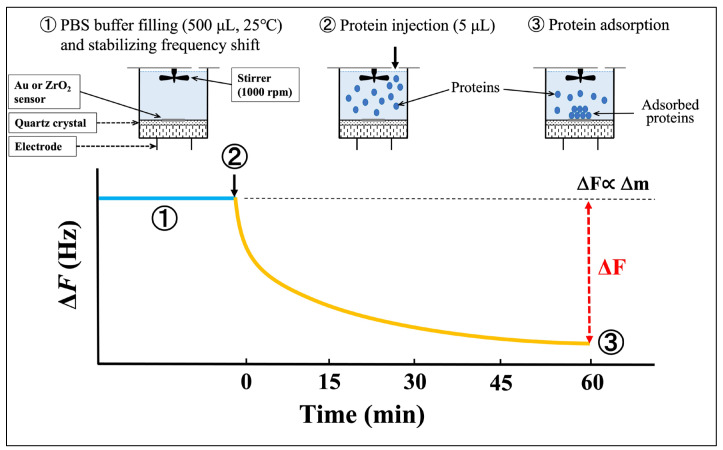
Schematic diagram of protein adsorption procedure and typical frequency-decrease curve. The blue line indicates the sensor stabilized after PBS injection, while the yellow line indicates the frequency decrease after protein injection.

A schematic diagram of the protein adsorption procedure and a typical frequency-decrease curve for the QCM measurements are shown in Figure 2. QCM is a mass-sensing technique based on a quartz crystal oscillator. The oscillation frequency decreases (Δ*F*) proportionally to the mass of the adsorbed material, such as a protein, when it binds to the sensor surface. For the 27 MHz oscillator used in the present study, a frequency decrease of 1 Hz corresponds to a mass increase of 0.62 ng/cm^2^. Therefore, the QCM sensor enables the detection of mass changes at the nanogram scale [32,33].

As described above, UV irradiation was used to clean each sensor before QCM measurement. With the sensor cell mounted on the socket, 500 μL of PBS was introduced into the cell using a micropipette. After the frequency shift stabilized, 5 μL of lactoferrin, peroxidase, or lysozyme solution was injected into the PBS in the cell. The resulting frequency decrease was monitored for 60 min following protein injection. The adsorbed amount (Δ*m*) of each protein at 60 min was calculated using Sauerbrey’s equation [34]:

$$∆F=-\frac{2F_{0}^{2}\;∆m}{A\sqrt{\rho_{q}\mu_{q}}}$$

where:

Δ*F*: Measured frequency shift (Hz);

Δ*m*: Mass change (g);

*F*_0_: Fundamental frequency of the quartz crystal (27 × 10^6^ Hz);

A: Electrode area (0.049 cm^2^);

*ρ*_q_: Density of quartz (2.65 g/cm^3^);

*µ*_q_: Shear modulus of quartz (2.95 × 10^11^ dyn/cm^2^).

The apparent reaction rate constant *k*_obs_ was determined by fitting the Δ*F* curve to the following time-dependent adsorption model:

$$∆F_{t}=∆F_{\infty }(1-e^{-k_{obs}\cdot t}).$$

where Δ*F*_∞_ is the frequency shift at infinite time.

*k*_obs_ reflects the combined effect of association and dissociation rates and represents the reciprocal of the relaxation time. The relaxation time is defined as the time required to reach 63% (=1 −e^−1^) of the saturated adsorption amount. Thus, a longer relaxation time corresponds to a slower adsorption rate, whereas a larger *k*_obs_ indicates faster adsorption.

Three runs of QCM measurements were performed.

### 2.5. Statistical Analysis

Statistical analysis was performed using the Statistical Package for the Social Sciences (SPSS Statistics 17.0; IBM Corp., Armonk, NY, USA). The significance level was set at *p* < 0.05. A non-paired *t*-test was used to compare the diameters of crystalline particles, surface roughness, and contact angles of Au and ZrO_2_ sensors. The adsorbed amounts and *k*_obs_ data from the QCM measurements were analyzed using one-way analysis of variance (ANOVA) followed by Tukey’s multiple comparison test. The results were expressed as the mean ± standard deviation (SD).

## 3. Results

### 3.1. Characterizations of Au and ZrO_2_ Sensors

**Figure 3:**
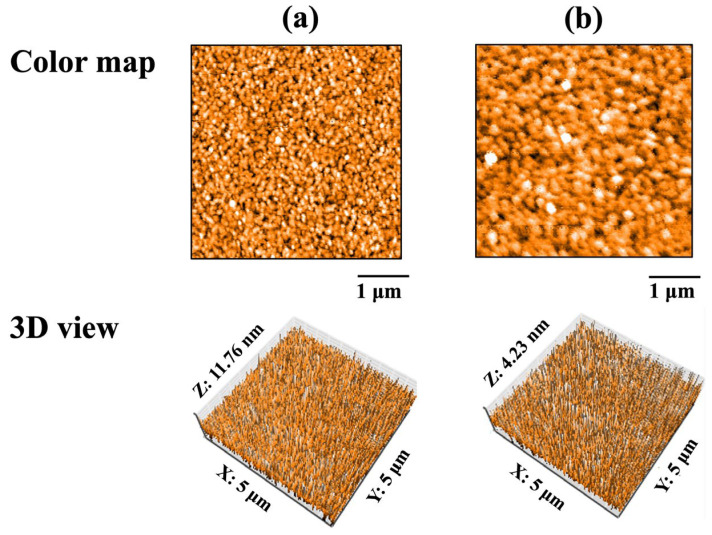
AFM images of QCM sensors before protein adsorption: (**a**) Au and (**b**) ZrO_2_.

AFM images of the QCM sensors before protein adsorption are shown in Figure 3. Spherical particles formed by sputtering were observed on the surfaces of Au and ZrO_2_ sensors. The average particle diameter on the Au sensor was 0.12 ± 0.04 μm, while that on the ZrO_2_ sensor was 0.20 ± 0.07 μm. The particle diameter on the ZrO_2_ sensor was significantly larger than that on the Au sensor (*p* < 0.05).

Table 1 lists the contact angles and surface roughness values of the Au and ZrO_2_ sensors before protein adsorption. No significant differences in surface roughness (Sa) were observed between the Au and ZrO_2_ sensors (*p* > 0.05). However, the Au sensor exhibited significantly larger contact angles than the ZrO_2_ sensor (*p* < 0.05). Therefore, the surface of the ZrO_2_ sensor was more hydrophilic than that of the Au sensor.

### 3.2. QCM Measurements

**Figure 4:**
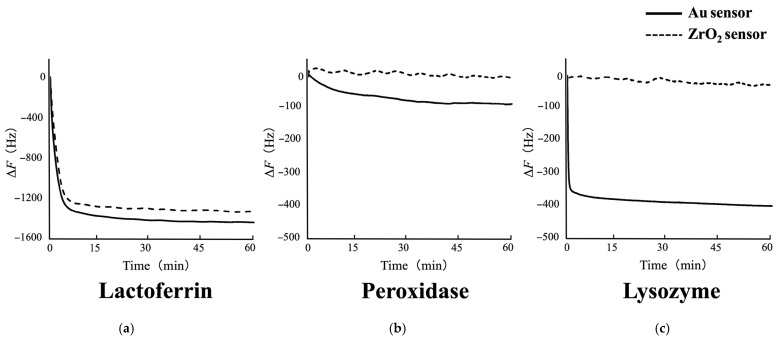
Typical Δ*F* curves for the adsorption of each salivary antimicrobial protein onto Au and ZrO_2_ sensors obtained from QCM measurements: (**a**) lactoferrin, (**b**) peroxidase, and (**c**) lysozyme. The adsorption process was monitored in real time throughout the measurements. The frequency decrease was recorded immediately after the injection of each protein. Lactoferrin adsorption resulted in a similar Δ*F* decrease on both sensors. Peroxidase and lysozyme caused a Δ*F* decrease on the Au sensor, while no change was observed on the ZrO_2_ sensor.

Figure 4 shows typical Δ*F* curves for the adsorption of each salivary antimicrobial protein onto the Au or ZrO_2_ sensor obtained from QCM measurements. A larger decrease in Δ*F* corresponds to a higher degree of protein adsorption on the sensor. The Δ*F* varied depending on the type of sensor and the adsorbed protein. The frequency decrease was recorded immediately after the injection of each protein. However, no decrease in Δ*F* was observed for the ZrO_2_ sensor when peroxidase and lysozyme were injected. In other words, the Au sensor exhibited a greater frequency decrease for peroxidase and lysozyme adsorption than the ZrO_2_ sensor. For lactoferrin adsorption, the frequency decrease did not differ depending on the sensor type.

**Figure 5:**
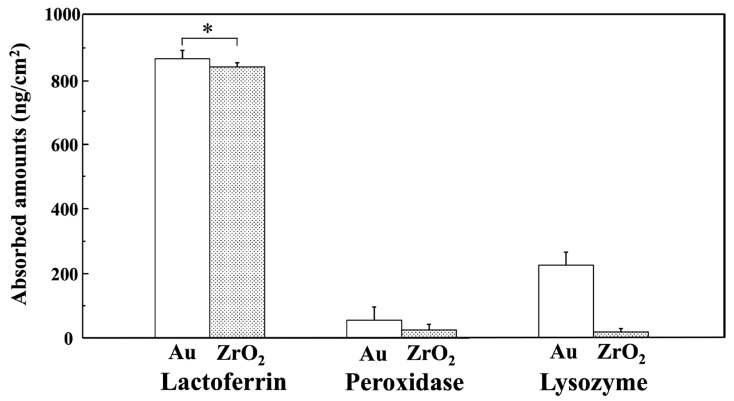
Estimated amounts of lactoferrin, peroxidase, and lysozyme adsorbed on Au and ZrO_2_ sensors 60 min after injection, calculated using the Sauerbrey equation. (* Connected bars: no significant difference (*p* > 0.05)). Lactoferrin adsorption on the Au and ZrO_2_ sensor was significantly higher than for peroxidase and lysozyme, and peroxidase and lysozyme showed significantly lower adsorption amounts on the ZrO_2_ sensor compared to the Au sensor (*p* < 0.05).

Figure 5 shows the estimated amounts of lactoferrin, peroxidase, and lysozyme adsorbed onto the Au and ZrO_2_ sensors 60 min after injection, as calculated using the Sauerbrey equation [34]. On the Au and ZrO_2_ sensors, lactoferrin displayed significantly higher adsorption than peroxidase and lysozyme (*p* < 0.05). A significant difference was absent between lactoferrin adsorption of the Au and ZrO_2_ sensors (*p* > 0.05). The amounts of peroxidase and lysozyme adsorbed on the ZrO_2_ sensor were significantly lower than those adsorbed on the Au sensor (*p* < 0.05).

**Figure 6:**
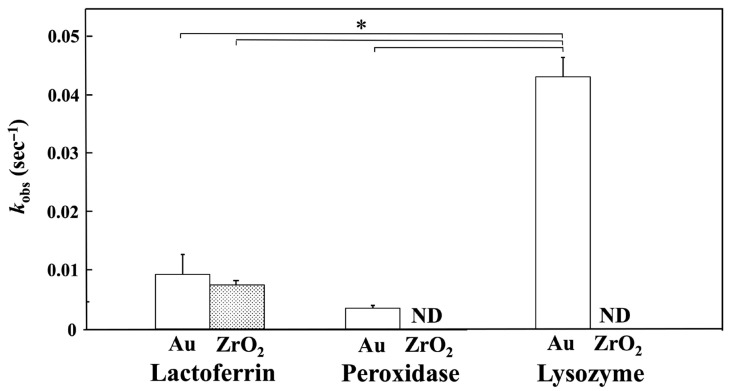
*k*_obs_ values for lactoferrin, peroxidase, and lysozyme adsorption onto Au and ZrO_2_ sensors obtained 10 min after injection by nonlinear fitting analysis. (* Connected bars: significant difference (*p* < 0.05); ND: not detected). The *k*_obs_ value for lysozyme on the Au sensor was significantly higher than those for lactoferrin on the Au and ZrO_2_ sensors and for peroxidase on the Au sensor (*p* < 0.05).

Figure 6 shows the *k*_obs_ values for lactoferrin, peroxidase, and lysozyme adsorption onto the Au and ZrO_2_ sensors obtained 10 min after injection, based on nonlinear fitting analysis. The *k*_obs_ values for peroxidase and lysozyme on the ZrO_2_ sensor were undetectable because curve fitting was not performed. The *k*_obs_ value for lysozyme on the Au sensor was significantly higher than those for lactoferrin on the Au and ZrO_2_ sensors and for peroxidase on the Au sensor (*p* < 0.05).

## 4. Discussion

This study investigated the adsorption behaviors of peroxidase, lactoferrin, and lysozyme onto ZrO_2_ surfaces using QCM analysis. The adsorption of these antimicrobial proteins on ZrO_2_ surfaces differed from that on Au surfaces. Au sensors, which are commonly used as metallic dental materials, were employed as controls for comparison with Au alloys.

Numerous methods exist for evaluating protein adsorption on materials, such as infrared reflection spectroscopy, ellipsometry, and surface plasmon resonance [35]. In this study, we used the QCM method, which enables real-time measurement of mass changes at the nanogram level. Additionally, a highly sensitive QCM device with a vibrational frequency of 27 MHz was utilized. Our previous studies have demonstrated the initial behavior of material implantation by accurately investigating the adsorption of various proteins using this QCM system [8,21,22,28,29,30].

An acquired protein film forms on tooth surfaces, and salivary proteins are adsorbed. Oral bacteria specifically bind to various salivary proteins and then form colonies [19]. In a previous study, we investigated the adsorption of bovine serum albumin and mucin onto denture base metals (Au, Ti, and Co–Cr alloy) using the QCM method [8]. We found that significantly lower levels of albumin were adsorbed onto Ti compared to Au and Co–Cr alloys. Au demonstrated significantly greater mucin adsorption than Co–Cr. In this study, we observed the adsorption behaviors of antimicrobial salivary proteins, which have the opposite effect on plaque formation. We conducted basic research to test the hypothesis that ZrO_2_ has low plaque adhesion. The present study showed no significant difference in the amounts of each protein adsorbed on the Au and ZrO_2_ sensors, which was an unexpected result. No direct correlation was observed between the adsorption of the antimicrobial proteins on ZrO_2_ compared to Au and plaque formation. However, a novel finding from this study was that differences were observed depending on the type of protein.

A protein concentration of 0.5 mg/mL has been primarily used in our previous QCM studies involving various proteins [21,22,28,29,30]. Human salivary albumin concentrations have been reported to range from 0.09 to 0.5 mg/mL or higher [36]. Because many of these studies focused on albumin adsorption, the same concentration was applied to lactoferrin, peroxidase, and lysozyme in this study to facilitate meaningful comparisons with past data.

No significant difference was observed in the surface roughness between the Au and ZrO_2_ sensors. However, the Au sensor was more hydrophobic than the ZrO_2_ sensor. A comparison of the protein adsorption amounts and *k*_obs_ rates of each protein onto the Au and ZrO_2_ sensors revealed either no significant difference or greater adsorption on the Au sensor. These results suggest that surface roughness and wettability have minimal impact on protein adsorption [37]. Electrostatic interactions should be considered as additional influencing factors. The isoelectric point (pI) of peroxidase is 7.3–7.5 [38], whereas those of lactoferrin and lysozyme are 8.2–8.9 [39] and 10.7 [40], respectively. Therefore, peroxidase was neutral, and lactoferrin and lysozyme were positively charged under the pH = 7.4 conditions used in this study. The zeta potential, an important surface electronic characteristic of materials, was measured as the basis for comparison. According to measurements using the streaming potential method [41], the zeta potentials of Au and ZrO_2_ at pH 7.4 are approximately −20 mV [8,42] and −43 mV [22,29,30], respectively. These results suggest that Au and ZrO_2_ surfaces were negatively charged under the conditions employed in this study. As a result, electrostatic attraction occurred between the positively charged proteins (lactoferrin and lysozyme) and the negatively charged sensors. Owing to stronger electrostatic attraction, a larger amount of lactoferrin was adsorbed than peroxidase on both sensors. Although lysozyme, like lactoferrin, is positively charged, it showed lower adsorption on both sensors compared to lactoferrin. However, the amount of lysozyme adsorbed and the adsorption rate on the Au sensor were higher than on the ZrO_2_ sensor. This may be related to the presence of S–S bonds in lysozyme, and possibly Au was chemically influenced and selectively adsorbed [43]. Other factors besides electrostatic interactions may have contributed to the adsorption of peroxidase and lysozyme onto the ZrO_2_ sensor. No significant difference was observed in the *k*_obs_ values of lactoferrin between the Au and ZrO_2_ sensors, which may be due to comparable electrostatic interactions with both surfaces. In contrast, the high *k*_obs_ value for lysozyme on the Au sensor likely reflects the contribution of S–S bonds, as discussed earlier. In practice, lysozyme adsorbs rapidly onto Au, while lactoferrin and peroxidase are presumed to adsorb subsequently. This difference in the adsorption sequence between Au and ZrO_2_ may influence subsequent plaque formation.

Nezu et al. [44] reported that lysozyme adsorbs most on Au surfaces, less on SiO_2_, and least on TiO_2_, with TiO_2_ adsorption occurring only at neutral pH by QCM-D (QCM with dissipation monitoring) analysis. Adsorption was reduced by salt, indicating that electrostatic interactions play a key role. ZrO_2_, like silica, is a ceramic material and may exhibit comparable properties such as plaque formation [45,46]. SiO_2_-based porcelain prostheses have been reported to show less accumulation of dental biofilm compared to metal prostheses. The markedly lower adsorption of lysozyme on ZrO_2_ relative to gold is consistent with the present findings and suggests that electrostatic interactions may play a significant role. Teichroeb et al. [47] reported in their QCM-D study that lysozyme adsorbs rapidly and reversibly onto polyHEMA surfaces, whereas lactoferrin adsorbs more slowly but forms a more stable layer. PolyHEMA, a key component of soft contact lenses, presents challenges for protein incorporation. When both proteins are present, they clarified that lactoferrin preferentially adsorbs and displaces lysozyme, thereby altering the overall adsorption behavior. The slower adsorption rate of lactoferrin compared to lysozyme was consistent with our study results. Thus, preferential adsorption of lactoferrin onto ZrO_2_ surfaces is expected.

We could not determine a clear relationship between the protein adsorption results and characterizations of the Au and ZrO_2_. Adsorption behavior is influenced by various factors, including the pH conditions of the environment. A PBS solution with a pH of 7.4 was employed in this experiment. Variations in pH may have influenced adsorption owing to biological reactions occurring in the oral environment, where bacterial biofilms form. The concentrations of lactoferrin, peroxidase, and lysozyme may represent another key factor affecting adsorption behavior. Changes in pH and concentration will be explored in future QCM studies. Additionally, proteins derived from saliva do not exist in isolation but interact with each other simultaneously. Therefore, measuring a mixture of multiple proteins is required for QCM analysis. Hirota et al. reported that fibronectin and albumin were adsorbed onto ZrO_2_ surfaces in two steps using QCM [30]. The proteins were added in two orders: fibronectin followed by albumin, and albumin followed by fibronectin. No significant difference was observed in the total amounts adsorbed between the two sequences. However, the *k*_obs_ of the second protein was significantly lower. This suggests that the first protein affects the rate of adsorption of the second one. These results indicate that in environments where multiple proteins coexist, adsorption behavior can be altered. Using this approach, the hypothesis that the order of protein adsorption differs between Au and ZrO_2_ can be evaluated, owing to the faster adsorption of lysozyme, potentially influencing plaque development. Further research is anticipated using sensors coated with materials other than Au and ZrO_2_. Additionally, this study suggests that controlled electrostatic interactions between material surfaces and antimicrobial proteins could contribute to the development of new dental devices with enhanced antimicrobial properties.

## 5. Conclusions

Our findings obtained through the QCM method demonstrate that the adsorption behaviors of peroxidase, lactoferrin, and lysozyme differed on Au and ZrO_2_ surfaces. The adsorption of lactoferrin onto the ZrO_2_ sensor was significantly higher than that of peroxidase and lysozyme. The adsorption of peroxidase and lysozyme onto the ZrO_2_ sensor was significantly lower than that onto the Au sensor. This study did not reveal a direct antimicrobial relationship through protein adsorption on Au or ZrO_2_. However, differences in adsorption behavior between the two materials were observed, highlighting the need to better replicate oral environmental conditions, such as pH and protein concentrations, in future experiments. In the oral cavity, various proteins interact with each other, and acid production by plaque or the presence of inflammation can lead to a decrease in pH, which may affect protein adsorption onto materials by altering their isoelectric points and influencing electrostatic interactions. Under these conditions, protein adsorption behavior may be altered, potentially influencing plaque formation and other biological responses. These differences may affect plaque formation, tissue healing processes, and bone osseointegration when using ZrO_2_. A deeper understanding of protein adsorption on ZrO_2_ may reveal its potential as a bio-functional material, contributing to the control of oral diseases. Future work will focus on analyzing mixed protein solutions and exploring sensors coated with materials other than Au and ZrO_2_.

## Figures and Tables

**Table 1:** Contact angle and surface roughness values of Au and ZrO_2_ sensors before protein adsorption.

Sensor	Contact Angle (°)	Surface Roughness (Sa, nm/25 μm^2^)
Au	30.94 (2.89) ^a^	2.23 (0.04) ^A^
ZrO_2_	8.22 (0.74) ^b^	2.07 (0.09) ^A^

The numbers in parentheses represent SD. Letters (a–x and A–X) indicate no significant difference (*p* < 0.05).

## Data Availability

The original contributions presented in the study are included in the article; further inquiries can be directed to the corresponding author.

## Abbreviations

The following abbreviations are used in this manuscript:

ZrO_2_	Partially stabilized zirconia
QCM	Quartz crystal microbalance
UV	Ultraviolet
AFM	Atomic force microscope
Sa	Surface roughness parameter (the 3D arithmetic height)
PBS	Phosphate-buffered saline
